# Chronotypes are significantly associated with emotional well-being in a general population cohort in Hungary

**DOI:** 10.1038/s41598-025-00893-8

**Published:** 2025-05-08

**Authors:** Anat Lan, Dora Torok, Haim Einat, Gyorgy Bagdy, Gabriella Juhasz, Xenia Gonda

**Affiliations:** 1 School of Behavioral Sciences, Tel-Aviv-Yaffo Academic College, Tel-Aviv, Israel; 2 NAP3.0 Neuropsychopharmacology Research Group, Semmelweis University, Budapest, Hungary; 3 Department of Pharmacodynamics, Semmelweis University, Budapest, Hungary; 4 Center of Pharmacology and Drug Research & Development, Semmelweis University, Budapest, Hungary; 5 Department of Clinical Psychology, Semmelweis University, Budapest, Hungary; 6 Department of Psychiatry and Psychotherapy, Budapest, Hungary

**Keywords:** Chronotype, Emotional well-being, Morningness-eveningness, Evening chronotype, Depression, Psychology, Human behaviour

## Abstract

Chronotypes reflect individual differences in the preference for wake and sleep times within a 24-hour period. They have been linked to emotional well-being, yet findings remain inconsistent, potentially due to variations in study populations, methodologies, and measures of emotional well-being. This study aimed to clarify the contribution of chronotypes to emotional well-being while controlling for demographic variables in a large cohort of 1,120 participants from the general population in Hungary. Emotional well-being was assessed using validated self-report measures, including the Brief Symptoms Inventory (BSI), Zung Self-Rating Depression Scale (ZSDS), State and Trait Anxiety Inventory-Trait (STAI-T), and Beck Hopelessness Scale (BHS). Chronotypes were determined using the Morningness-Eveningness Questionnaire (MEQ). Comprehensive stepwise regression analyses were performed to evaluate relationships between demographic factors, chronotypes, and emotional well-being. Statistical analyses revealed that individuals with evening chronotypes were more likely to report lower emotional well-being, even after adjusting for demographic factors such as age, gender, and socioeconomic status. Regression models demonstrated the unique contribution of chronotypes to emotional well-being, highlighting the vulnerability of evening types. These findings underscore the importance of considering chronotypes in understanding emotional health and suggest potential directions for chronotype-specific interventions to promote mental well-being.

## Introduction

The relationship between chronotypes and emotional well-being is an intriguing area of study within the fields of chronobiology and psychology. Chronotypes refer to an individual’s natural predisposition to be more alert and active during specific times of the day, often categorized as morning (morning chronotype), evening (evening chronotype), or in between (intermediate chronotype)^1^. These chronotypes are closely tied to the individual’s circadian rhythm, and can have a profound impact on mood, emotions, and overall well-being^1,2^.

Morning chronotypes tend to feel most alert and positive in the earlier hours of the day. They often experience a better mood in the morning, which can be at least partially attributed to the alignment of their innate circadian rhythm with the typical diurnal cycle^1^. Morning chronotypes may feel more proactive and optimistic, and their synchronization with the external world’s schedule may at least in part explain these feelings^3^. In contrast, evening chronotypes, or “night owls,” experience their peak alertness and positive affect during the evening and nighttime hours. These individuals often struggle with early morning activities and may experience morning irritability as they attempt to function during a time that is not synchronized with their internal clock^4,5^. They also more significantly have to face “social jetlag” resulting from a significant discrepancy between their sleep-wake schedules and the expectations from society and its work and activity schedules, contributing to irregular sleep-wake cycles, loss of sleep during weekdays and catching up with sleep on weekends^6^.

Chronotypes are distributed in the population with a near-normal distribution, somewhat skewed towards evening chronotypes^7^. Most of the population are categorized with intermediate chronotype representing a balance between morning and evening preferences. Intermediate chronotypes can generally maintain a moderate level of alertness and positive affect throughout the day^7^.

The relationship between chronotypes and affect extends beyond daily mood fluctuations. Research has shown that a misalignment between an individual’s chronotype and their daily schedule can lead to a phenomenon known as social jetlag^8^. Social jetlag occurs when people with different chronotypes are forced to adhere to a common schedule, such as fixed morning start times for work or school, which may not align with their biological preferences. This misalignment has been linked to reduced well-being, heightened stress, and negative effects on overall mental and physical health^9,10^. This issue is particularly relevant for evening chronotypes, who often face chronic sleep deprivation due to morning commitments, exacerbating mood and cognitive challenges^11^.

Understanding the relationship between chronotypes and emotional well-being has significant implications for various aspects of daily life. It can inform decisions about work schedules, social activities, and even clinical interventions for mood disorders^12,13^. Tailoring daily routines to better align with an individual’s chronotype, or other circadian-related interventions such as sleep hygiene education or managing light exposure schedules, can enhance overall well-being and improve mental and physical health^14^.

Despite the large body of evidence regarding the relationship between chronotypes and well-being, data is still inconsistent. One explanation for the inconsistency is that the effects of chronotypes might be dependent on the specific sample of participants in the different studies^15^, with different significance in dissimilar age groups, genders, physical environments, and other demographic factors^16^. Given the inconsistencies in previous findings, which often stem from variations in study populations, methodologies, and measures, the present study seeks to address these gaps by employing a large, demographically diverse cohort and validated self-report measures. This approach allows for a more comprehensive evaluation of the relationship between chronotypes and emotional well-being, independent of demographic confounders. By analyzing data from a large cohort of participants from the general population in Hungary, this research seeks to enhance our understanding of how chronotypes relate to emotional well-being, potentially informing future interventions aimed at improving mental health. To clarify, we use the term emotional well-being in this study to refer to the current emotional state of participants, as reflected by levels of psychological distress and life satisfaction. The composite score we created, and described below in the methods section, is based on standardized scores from validated questionnaires that assess symptoms of depression, anxiety, hopelessness, and general psychological distress.

## Results

The study included 1,121 participants who provided self-reported demographic and health data. Of the participants, 774 were females and 347 were males. The mean age was 31.5 years (± 0.3 SEM), with a range of 18 to 60 years. Marital status distribution showed 530 participants as married or cohabiting, 501 as single, 74 as divorced or separated, 11 as widowed, and 5 with no data. Regarding parenthood, 815 participants reported having no children, while 296 reported having children and 10 did not report. Financial situation was self-rated as bad by 65 participants, fair by 319, and good by 729 and 8 did not report. A history of psychiatric or emotional problems was reported by 424 participants, while 697 reported no such history. General health status was rated as poor by 35 participants, fair by 297, good by 604, and excellent by 184 and 1 did not report.

To create a single composite measure of emotional well-being, Z-scores were calculated based on four questionnaires: Brief Symptoms Inventory (BSI), Zung Self-Rating Depression Scale (ZSDS), State and Trait Anxiety Inventory-Trait component (STAI-T), and Beck Hopelessness Scale (BHS). These Z-scores were then averaged to compute the Emotional Well-Being (EWB) composite score. These measures, while assessing different domains of emotionality, were highly correlated (e.g., BSI and ZSDS: *r* = 0.72, *p* < 0.001; ZSDS and STAI-T: *r* = 0.85, *p* < 0.001). Higher Z-scores indicated lower emotional well-being (Table 1).

**Table 1:** Correlations between separate measures of emotional well-being.

	ZSDS	STAI-T	BHS
BSI	*r* = 0.72*	*r* = 0.75*	*r* = 0.55*
ZSDS		*r* = 0.85*	*r* = 0.62*
STAI			*r* = 0.59*

* Symbolizes *p* < 0.001.

Demographic and health factors were analyzed for their association with emotional well-being (Table 2). Gender differences showed better emotional well-being among males compared to females (t(1106) = 4.61, *p* < 0.0001, Cohen’s d = 0.3). Age was weakly correlated with emotional well-being but was not significant (*r* = 0.07, *p* = 0.02). Marital status was a significant factor, with married or cohabiting individuals reporting better emotional well-being than single or divorced/separated individuals [F(3,1099) = 15.4, *p* < 0.0001, Cohen’s d for married/cohabiting vs. divorced/separated = 0.61]. Participants with children showed slightly better emotional well-being than those without children (t(1092) = 3.92, *p* < 0.0001, Cohen’s d = 0.12). Financial status was strongly associated with emotional well-being, with better financial situations correlating with higher scores [F(2,1097) = 42.2, *p* < 0.0001, Cohen’s d for good vs. bad = 0.86]. Participants with a history of psychiatric disorders reported significantly lower emotional well-being compared to those without such a history (t(1106) = 15.05, *p* < 0.0001, Cohen’s d = 0.90). Better general health was also associated with better emotional well-being [F(3,1103) = 124.2, *p* < 0.0001, Cohen’s d for excellent vs. poor = 2.43].

**Table 2:** Associations of demographic and health factors with emotional well-being.

Variable	Association with emotional well-being	Statistics
Gender	Emotional well-being of males better than of females	t(1106) = 4.61, *p* < 0.0001 [Cohen’s d = 0.3, small].
Age	Emotional well-being does not change with age	*r* = 0.07, *p* = 0.02 (N.S.).
Marital status	Emotional well-being of individuals who are married or cohabiting with partner is better than in individuals who are single and both are better off compared with individuals who are divorced or separated.	ANOVA: F(3,1099) = 15.4, *p* < 0.0001; post-hoc: married/cohabiting > single > divorced/separated [Cohen’s d: Married/cohabiting vs. Divorced/separated d = 0.61, medium].
Children	Individuals with children show better emotional well-being compared with individuals without children.	t(1092) = 3.92, *p* < 0.0001 [Cohen’s d = 0.12, very small].
Financial status	Better financial situation (as per the pre-defined groups), is associated with better emotional well-being.	ANOVA: F(2,1097) = 42.2, *p* < 0.0001; post-hoc: Good > Fair > Bad [Cohen’s d: Good vs. Bad = 0.86, large].
History of psychiatric disorders	Individuals with psychiatric history report lower well-being compared with individuals without psychiatric history.	t(1106) = 15.05, *p* < 0.0001 [Cohen’s d = 0.90, large].
General health status	Better general health is associated with better reported emotional well-being.	ANOVA: F(3,1103) = 124.2, *p* < 0.0001. Excellent > Good > Fair > Poor [Cohen’s d: Excellent vs. Poor = 0.2.43, very large].

**Fig. 1:**
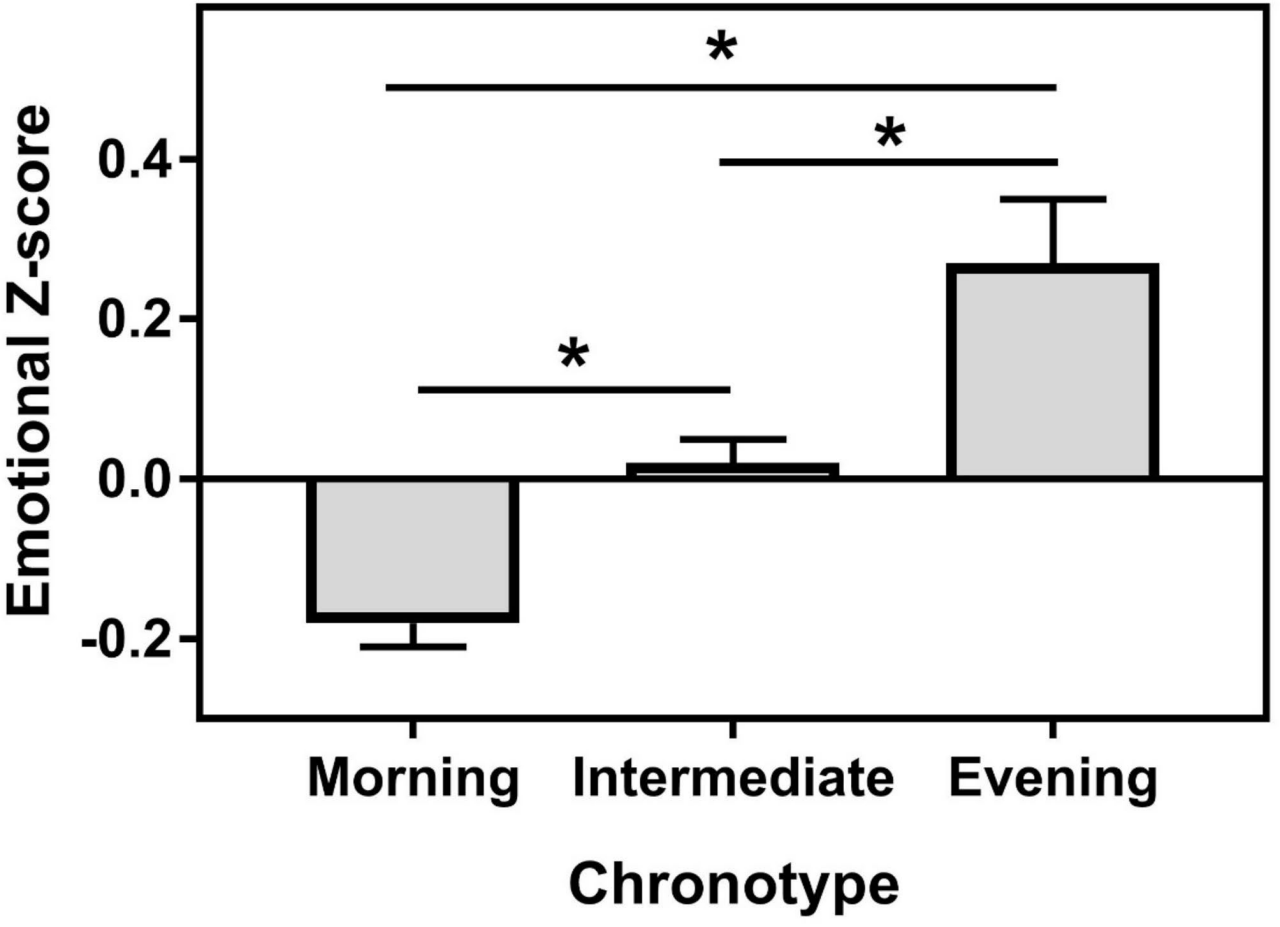
Relationship between chronotypes and aggregate score for emotional well-being. * indicates *p* < 0.05.

Chronotype distribution, assessed using the Morningness-Eveningness Questionnaire (MEQ) score, followed a normal distribution (χ2(7) = 20.6, *p* = 0.005) with 202 morning type, 788 intermediate type and 130 evening type (data were missing for one participant). Chronotype was significantly associated with emotional well-being [F(2,1104) = 7.7, *p* = 0.0005]. Post-hoc analyses (LSD) showed that morning chronotypes had better emotional well-being than intermediate chronotypes (*p* = 0.006, Cohen’s d = 0.22) and evening chronotypes (*p* = 0.0002, Cohen’s d = 0.44). Intermediate chronotypes also scored better than evening chronotypes although the difference did not reach statistical significance (*p* = 0.024, Cohen’s d = 0.22) (Fig. 1). To further assess possible interaction between gender and chronotype we conducted a 2-way ANOVA with EWB as dependent factor, Gender and Chronotype as independent factors. The results of this ANOVA indicate close to significant effects of Gender [F(1,1090) = 5.0, *p* = 0.025] and Chronotype [F(2,1090) = 4.71, *p* = 0.009], but no interaction [F(2,1090) = 0.67, *p* = 0.5].

Regression analysis examined the partial contribution of chronotypes to emotional well-being beyond demographics and health factors. The overall model was significant (R² =0.352, *p* < 0.0001), and chronotype contributed uniquely (R²=0.077, *p* = 0.002). Key predictors included gender (β=−0.07, *p* = 0.004), marital status (β = 0.09, *p* < 0.001), financial situation (β=−0.12, *p* < 0.001), psychiatric history (β = 0.26, *p* < 0.001), and general health (β=−0.35, *p* < 0.001). Chronotype (β = 0.08, *p* = 0.002) remained significant in this model even with the inclusion of other factors (Table 3).

**Table 3:** Regression analysis with EWB as the dependent variable.

Predictors	β	SE(β)	b	SE(b)	t(1080)	*p*-value
Intercept			1.42	0.20	6.97	< 0.001
Gender	−0.07	0.02	−0.14	0.05	−2.88	0.004
Marital status	0.09	0.03	0.12	0.04	3.41	< 0.001
Child	−0.05	0.03	−0.11	0.05	−2.00	0.045
Finance	−0.12	0.03	−0.19	0.04	−4.85	< 0.001
History of psychiatric disorders	0.26	0.03	0.49	0.05	10.03	< 0.001
General health status	−0.35	0.03	−0.44	0.03	−13.23	< 0.001
Chronotype	0.08	0.02	0.13	0.04	3.10	0.002

Because the current study was mostly designed to explore the effects of chronotype in non-clinical population we performed the analysis again after exclusion of participants who indicated past or present psychiatric disorders. The number of participants in this analysis was 689. The results for the smaller cohort were qualitatively similar to the results for the entire cohort. In the ANOVA analysis chronotype had a significant effect on the combined emotional well-being parameter [F(2,682) = 7.87, *p* = 0.0004; post hoc: Evening chronotype > Intermediate chronotype (*p* = 0.006), Intermediate chronotype > Morning chronotype (*p* = 0.02), and Evening chronotype > Morning chronotype (*p* = 0.0001)]. The regression analysis further supported the contribution of chronotype to the compound measure of emotional well-being (R²=0.12, *p* < 0.001).

## Discussion

The current study offers a comprehensive examination of emotional well-being in non-clinical populations, addressing the multifaceted influence of chronotypes on various emotional domains. Emotional well-being encompasses a wide range of feelings, cognitions, moods, and behaviors. Circadian rhythms have been implicated in these processes, and the role of chronotypes has gained significant attention. While studies often report a disadvantage for evening chronotypes compared to morning and intermediate chronotypes^5,15^, findings across the literature remain inconsistent, particularly in non-clinical populations.

One of the main challenges in the field is the variability in tools used to assess emotional well-being. Different studies utilize different instruments, leading to inconsistencies in findings. For example, a study by Tzischinsky and Shochat^4^ found relationship between chronotypes, mood and subjective quality of life in a cohort of middle school students. The tools used in this study were the modified School Sleep Habits Survey and the Pediatric Quality of Life Inventory Short Form. Another study in a larger cohort of teenagers (*n*= 1600) also reported that evening-type adolescents show lower psychological well-being, compared with morning or intermediate chronotypes^17^. The tools used in this study included the Adolescents Veçu et Santé Perçue de L’adolescent (VSP-A) and the Morningness-Eveningness Scale for Children (MESC). In contrast, Selvi and colleagues did not find direct connections between chronotypes and suicidal ideation in healthy controls^18^. This study used Beck Depression Inventory, MEQ, Suicide Ideation Scale and Suicidality section of the Mini-International Neuropsychiatric Interview. Also, in a study in nursing students during the COVID-19 epidemy, data did not show significant relationship between chronotypes and depression symptoms or quality of life although it is possible that there was some influence of chronotypes that was mediated through social jetlag. The study used Center for Epidemiological Studies Depression Scale, and World Health Organization Quality of Life Scale^19^. Another study in office workers did not find relationship between chronotypes and mental well-being using the Mental component summary from the RAND 12 items health survey^20^. Yet, a study by Levandovski and colleagues showed a three-way relationship between chronotypes, social jetlag and depression in a large cohort of rural population in Brazil^21^. Altogether, data is incongruent, and the tools used to assess emotional well-being vary greatly across studies.

The current study addresses this issue by employing a composite measure of emotional well-being. By integrating multiple validated tools—each assessing distinct emotional dimensions – and applying Z-score transformations, we aimed to provide a holistic understanding of emotional well-being. This approach, of utilizing Z-scores to consolidate related measures and construct a comprehensive variable, is not novel; it has been employed in both animal and human research, including in studies related to emotion. For example, Guilloux and colleagues employed Z-score transformations to amalgamate multiple measures within a specific behavioral dimension in their mouse models. Their findings indicated that this combined measure reduced the variability observed in individual tests and provided a clear characterization of emotional traits in the mice^22^. Likewise, a study conducted in rats used combined Z-scores from two distinct behavioral tests to elucidate the impact of environmental interventions on emotional behavior^23^. Similar practice was used in human studies. For example, Raymond and colleagues employed Z-score transformations to create a socio-emotional composite score derived from various anxiety- and depression-related questionnaires^24^. Likewise, a recent small study demonstrated the effects of chronotypes on a combined score of several questionnaires related to mood and anxiety^5^. The strength of generating a composite measure lies in the power of such variable to shed light on the factors that are associated with emotional well-being including chronotypes.

Our findings align with the broader literature indicating that evening chronotypes face greater challenges in various aspects of life, including emotional well-being^5,25,26^.However, some studies also show few advantages for evening chronotypes such as possibly higher emotional intelligence^27^. Importantly, many prior studies focused on clinical populations, whereas our study extends these findings to a large cohort of non-clinical participants. Some previous work examined also the effects of chronotypes on well-being in non-clinical cohorts. The general outcomes of these studies are in line with the current findings and demonstrate reduction in variety of domains of well-being in evening chronotypes (Whittman et al., 2009; Gulec et al., 2012^28,29^. The distinction between clinical and general populations is crucial, as it highlights the relevance of chronotypes beyond clinical settings and emphasizes their impact on everyday emotional experiences. The current study adds clear information on the effects of chronotype on emotion in a large cohort of the general population using tools that are more appropriate for non-clinical participants.

While this study provides valuable insights, several limitations should be acknowledged. First, we used a general population sample, recruited through general practices and advertisements, We did not aim to recruit a representative sample. Yet our recruitment process inherently may have introduced certain biases which should be considered when interpreting and generalizing our results. Second, the composite measure of emotional well-being assumes equal weighting of its components, which may not fully capture the nuanced contributions of individual variables. However, the strong correlations observed among the original variables suggest that the overall findings are robust. Thus, it is reasonable to claim that even with altered weights for the individual variables in the composite “emotional well-being” variable, the link with chronotypes would persist. Additionally, the focus of the study was to evaluate the effects of chronotypes on emotional well-being of healthy participants, but the selection of participants did not exclude individuals with previous or current mental disorders. To overcome this limitation, we analyzed separately participants who did not indicate past or present psychiatric disorder. As shown in the results section, the separate analysis of the individuals with no psychiatric history did not differ from the results of the entire cohort.

One more limitation is that the study did not control for the time of day when participants responded to the questionnaires. Previous studies clearly demonstrated that time of day affects various emotional and cognitive variables and interacts with chronotype in such measures^30,31^.

Altogether, the current study clearly supports the notion that chronotypes have a significant contribution to emotional well-being not just in the context of psychiatric populations but also in mentally healthy population. This knowledge leads us to the next question which is how can we device chronotherapy interventions targeting large populations to assist individuals with evening chronotype overcome their challenge and achieve better emotional well-being.

## Methods

### Participants

The present study was performed in the Hungarian cohort of a general population sample which is part of the NewMood study (New Molecules in Mood Disorders, Sixth Framework Program of the EU, LSHM-CT-2004–503474) funded by the European Union. Participants of European white ethnic origin between 18 and 60 years were recruited through advertisements and general practices in Budapest, Hungary. Participation was voluntary, and participants did not receive any reimbursement for taking part in the study. A detailed description of the recruitment method, inclusion and exclusion criteria, and the sample can be found in our previous papers^32^. All participants provided written informed consent prior to participating in the study. All procedures were carried out in accordance with the Declaration of Helsinki and were approved by the Scientific and Research Ethics Committee of the Medical Research Council, Budapest, Hungary.

## Procedure

After agreeing to participate, participants filled out the NewMood questionnaire pack, including a detailed background questionnaire focusing on demographic information and mental and somatic health-related data, as well as a series of psychometric instruments assessing different aspects of psychological well-being, including emotional distress, depressive symptoms, anxiety, and hopelessness.

## Tools

*Demographic and background questionnaire*: This questionnaire collected information regarding gender (female, male, or other), age (in years), marital status (married/cohabiting, single, divorced/separated/widowed), children (yes/no), financial status (good, fair, bad), history of psychiatric disorders (yes/no), and general health (excellent, good, fair, bad).

*Brief symptoms inventory (BSI)*: The BSI is a widely used self-report questionnaire designed to assess psychological distress and symptoms of psychopathology^33,34^. It provides a quick and reliable evaluation of an individual’s mental health by measuring current psychological symptoms and distress. The BSI consists of 53 items that cover a range of symptoms, including anxiety, depression, somatization, and interpersonal sensitivity. Respondents rate the extent to which they have been bothered by these symptoms over the past week on a 5-point Likert scale, ranging from “Not at all” to “Extremely.” The BSI yields several summary scores, including the Global Severity Index (GSI), which offers an overall measure of psychological distress. The Hungarian version of the BSI has been validated in prior studies^35^.

*Zung Self-Rating Depression Scale (ZSDS)*: The ZSDS assesses the severity of depressive symptoms in individuals^36^. The scale consists of 20 items rated on a 4-point Likert scale (ranging from “None or a little of the time” to “Most or all of the time”), covering emotional, cognitive, and physical symptoms associated with depression. The Hungarian version of the ZSDS was previously validated^37^.

*State and Trait Anxiety Index (STAI)*: The STAI is a widely used psychological assessment tool that is designed to measure state and trait anxiety^38^. This self-report questionnaire consists of two separate 20-item scales. The current study utilized the trait component (STAI-T). Respondents rate their feelings on a 4-point Likert scale ranging from “Not at all” to “Very much.” The Hungarian version of the STAI was validated previously^39^.

*Beck Hopelessness Scale*(BHS): The BHS measures three major aspects of hopelessness: feelings about the future, loss of motivation, and expectations^40,41^. It consists of 20 true-false items and is designed for adults. The Hungarian version of the BHS was previously validated^42^.

*Morningness-Eveningness Questionnaire (MEQ)*: The MEQ is a self-report questionnaire designed to assess an individual’s chronotype, which is their natural preference for being active and alert during specific times of the day^43^. The standard MEQ includes 19 multiple-choice questions, with scores indicating “evening types” (41 and below), “morning types” (59 and above), and “intermediate types” (42–58). The Hungarian version of the questionnaire was previously validated^11^.

### Statistical analysis

Data for BSI, ZSDS, STAI-T, and BHS were transformed using Z-scores with the formula:

$$Z_i = \frac{X_i - \bar X}{{\rm STD} X}$$

Z-scores were arranged so that higher scores represent lower emotional well-being (more symptoms). The Z-scores for these variables were then averaged to generate a single variable referred to as “emotional well-being” (EWB). T-tests, ANOVAs, and Pearson’s correlations were used to evaluate possible relationships between demographic factors, chronotypes, and EWB. Variables showing an initial indication of a relationship with EWB were included in a comprehensive stepwise regression analysis to explore their partial contributions. Statistical analysis was performed using Statistica 13.0 (TIBCO Software Inc.). A similar method was recently employed in a study related to chronotypes and emotional well-being in a different population^5^. After applying the Bonferroni correction for multiple comparisons, the statistical significance threshold was set at *p* ≤ 0.006.

## Data Availability

The datasets generated and analysed during the current study are available in the FigShare repository, with doi 10.6084/m9.figshare.28106201.

## Data Availability

The datasets generated and analysed during the current study are available in the FigShare repository, with doi 10.6084/m9.figshare.28106201.
